# The need for a Rehabilitation Model to address the disparities of public healthcare for people living with HIV in South Africa

**DOI:** 10.4102/ajod.v4i1.137

**Published:** 2015-06-08

**Authors:** Verusia Chetty, Jill Hanass-Hancock

**Affiliations:** 1 Discipline of Physiotherapy, University of KwaZulu-Natal, South Africa; 2 Health Economics and HIV and AIDS Research Division, University of KwaZulu-Natal, South Africa

## Abstract

Rehabilitation in the context of HIV management in Africa is still a neglected field which holds great promise for the improvement of the quality of life as well as integration of people living with HIV back into their communities and homes. However, rehabilitation has not been incorporated into HIV care despite the fact that a large number of people living with HIV experience disability. The dearth of literature and lack of models of care to roll out rehabilitation for people living with HIV in Africa are astounding. Well-resourced countries have emerging approaches on the management of disability in the context of HIV. However, epidemic countries are still lacking such an approach neglecting the devastating effects of disability on individual livelihoods and antiretroviral treatment adherence. Thus, rehabilitation needs to be integrated into the response to HIV. This article advocates for the development and implementation of a model of care to guide rehabilitation of people living with HIV in South Africa.

## Introduction

South Africa is the epicentre of the global HIV epidemic with more than 6.4 million people living with HIV in this part of the world (Shisana *et al.* 2014). Although the country shows initial successes in reduction of HIV incidence, the overall number of people living with HIV will still rise in years to come. For instance, the 2012 Human Sciences Research Council (HSRC) household survey reveals that HIV prevalence increased from 10.6% in 2008 to 12.3% in 2012 with 2 million people on antiretroviral treatment (ART) (Shisana *et al.* 2014). With the up scaling of the ARTs people are now surviving, however they experience new challenges related to a life with chronic illness which may include disablement related to HIV, its co-morbidities and their treatments (Hanass-Hancock, Regondi & Nixon 2013; Meintjies *et al.* 2012; Nixon *et al.* 2011a). A recent scoping review on HIV-related disability in hyper-endemic countries revealed that people living with HIV experience a range of impairments affecting the body function (mental, sensory, cardiovascular, respiratory, digestive, metabolic, reproductive and muscle functions), activity and participation levels leading to disability (Hanass-Hancock *et al.* 2013). These disabilities impact quality of life, livelihoods and adherences to ARTs and provide an increased burden to health care (Cobbing *et al.* 2013; Hanass-Hancock *et al.* 2013). Consequently, adherence to ARTs is becoming the focus of attention in health care research as great investment is put into South Africa to roll-out ARTs. Mental health impairments and its disabling effects on people living with HIV directly impact adherence and pose a threat to health care (Petersen *et al.* 2014). Furthermore, unemployment of people living with HIV affects adherence to ARTs as people cannot afford treatment and being unemployed may also result in depression which has a ripple effect on adherence to treatment regimen (International Labor Organisation Report 2013). Emerging literature argues that HIV, like other chronic diseases, needs to be accompanied by a continuum of care including rehabilitation and mental health services (Cobbing *et al.* 2013; Hanass-Hancock *et al.* 2013; Nixon *et al.* 2011b). However, in Africa there is a gap of conceptualising HIV as a chronic disease that involves disability and the development and implementation of rehabilitation approaches that are feasible and prevent or reduce the disabling effects of living with HIV.

Rehabilitation professionals in Africa (Chetty & Maharaj 2013; Cobbing *et al.* 2013; Hanass-Hancock *et al.* 2012; Hughes *et al.* 2004; Jelsma *et al.* 2002) argue that there is a need for rehabilitation within health care systems to offer a continuum of coordinated, multi-levelled, multi-discipline and evidence-based service to address the dynamic nature of the disease. However, there has been no consensus related to the extent to which rehabilitation approaches or strategies have been effectively integrated into HIV management in the general context of health nor has there been discussion related to what strategies or approaches to rehabilitation would be more feasible in a holistic model of HIV care in a country like South Africa (Cobbing, Hanass-Hancock & Deane 2014).

In South Africa, rehabilitation of people living with HIV differs from the public to the private sector. There is a disparity with regard to resources available to individuals accessing the public health sector compared to individuals who can afford private care. Public health care lacks the infrastructure and funding to manage the health care demands of the large number of people accessing its services and this is confounded by poor governance and shortages of health care workers (World Health Organisation Bulletin 2015). For the purposes of this article, emphasis is maintained on rehabilitation offered within the public health sector. The article provides an overview of the current models of rehabilitative care in different settings and discusses how these can inform the inclusion of rehabilitation into a model of care for people living with HIV within a public health care South African framework.

### Ethical clearance

Full ethical clearance to conduct this PHD research in Health Science's under the supervision of Dr Jill Hanass Hancock. (Ethical clearance no. HSS/1319/012D). The protocol submitted is a PHD in Health Sciences (University of KwaZulu-Natal).

### Emerging evidence of rehabilitation in the context of HIV

The World Health Organization's International Classification of Functioning, Disability and Health (ICF) has changed the disability paradigm from unilateral into multidimensional, in that disability is not only seen to affect an individual's body but their social being as well (World Health Organisation 2002). The interactions between health conditions, intrinsic contextual features of the individual and extrinsic contextual features of the social and physical environment make this framework suitable to understand the novel challenges posing resource limited settings such as South Africa. The ICF framework has lent itself to studies in a South African context (Hanass-Hancock *et al.* 2013), which allows for better understanding of HIV, disability and rehabilitation (Myezwa *et al.* 2009, Van As *et al.* 2009).

Worthington *et al.* (2005) used qualitative means to develop an insightful rehabilitation framework to improve the service for people living with HIV in Canada. This HIV conceptual rehabilitation framework was developed in consultation with various stakeholders including people living with HIV and rehabilitation professionals. It offered a broader understanding of rehabilitation including psychological, social and vocational dimensions but remained client-focused and goal oriented. The rehabilitation framework took route in the ICF which propagates rehabilitation as a ‘dynamic process, including all prevention and/or treatment activities and/or services that address body impairments, activity limitations and participation restrictions for an individual’ (Worthington *et al.* 2005). Worthington *et al.* (2009) explored and developed the rehabilitation needs of people with HIV living in Canada through a national survey of health professionals as providing tools and support to do what is meaningful to them. These tools extend beyond health care and include vocational and fiscal support in addressing the rehabilitation needs of people living with HIV (Worthington *et al.* 2009).

As an imperative in the rehabilitation of people living with HIV, the authors of this article identified three concepts to be included in the rehabilitation framework for a South African setting bearing in mind the ICF and Worthington *et al.*’s (2005, 2009) contribution into understanding disability and rehabilitation. Firstly, the setting in which rehabilitation occurs needs to address varying degree of demands on resources and rehabilitation services that is available and this must be tailored into a rehabilitation framework (New South Wales Department of Health 2010). Secondly, people living with HIV require different levels of care and rehabilitation at different points in their life. Disability may also be experienced episodically and this will impact the service delivery as people living with HIV may experience shifting levels of disablement and require more or less rehabilitation intervention depending on their needs at a point in time of care (O’ Brien *et al.* 2011). Thirdly, the flow of people living with HIV may include the movement from the acute care setting to the sub-acute care setting and from the sub-acute care setting back into the community and home (New South Wales Department of Health 2010) and a rehabilitation framework needs to ensure that these links work efficiently.

Community-based rehabilitation which utilises local resources in areas with limited infrastructure (Iemmi *et al.*, 2014) and home-based care taking rehabilitation to people living with HIV are two working rehabilitation approaches in South Africa. These approaches are well suited contextually taking into consideration lack of resources but still there lacks a model of care that brings together these existing practices and approaches.

Canada is amongst the leading countries addressing rehabilitation of people living with HIV and for over 15 years has mobilised a working group of stakeholders forming the Canadian Working Group on HIV and Rehabilitation (CWGHR 2013). CWGHR has established pristine educational material informing the rehabilitation of people living with HIV in Canada some of which is being adapted with contextual variance in sub-Saharan Africa to inform and aid in rehabilitation practice (Nixon *et al.* 2014). The module proposes to bridge the existing knowledge gap with regard to rehabilitation at a local level in low- to middle-income contexts. Adapting and developing such guidelines will aim to offer a feasible approach of providing holistic and multidisciplinary service for people living with HIV in these settings. For instance, a discussion around task-shifting and usage of lay personal to deliver rehabilitation may not be necessary in the Canadian context but might be one of the few feasible approaches to include in rehabilitation in the context of resource poor settings such as South Africa.

### Elements in Developing a Model of Care in the Context of Rehabilitation

A Model of Care ‘is a multifaceted concept, broadly defining the way in which health care is delivered including the values and principles; the roles and structures; and the care management and referral processes. Where possible the elements should be based on best practice evidence and defined standards and provide structure for the delivery of health services and a framework for subsequent evaluation of care’ (Davidson *et al.* 2006; Queensland 2000). Many shortfalls in the delivery of care in varied health settings such as poor infrastructure lend to the development of novel models by health care professionals as they respond to these demands on health care services (Davidson *et al.* 2006).

These shortfalls often promote a convergence between research and the health care setting (Davidson *et al.* 2006). In well-resourced countries such as Australia this has led to the development and implementation of models of care in rehabilitation of patients with various conditions. These include cardiac, orthopaedic, neurological fields as well as high impact conditions like amputees (New South Wales Department of Health 2010; South Australia Department of Health 2011a; South Australia Department of Health 2011b; Western Australia Department of Health 2007; Western Australia Department of Health 2008). During evaluation of the development of these models a number of strategies have been identified as crucial for a meaningful process and development of a working model in rehabilitation.

**FIGURE 1:**
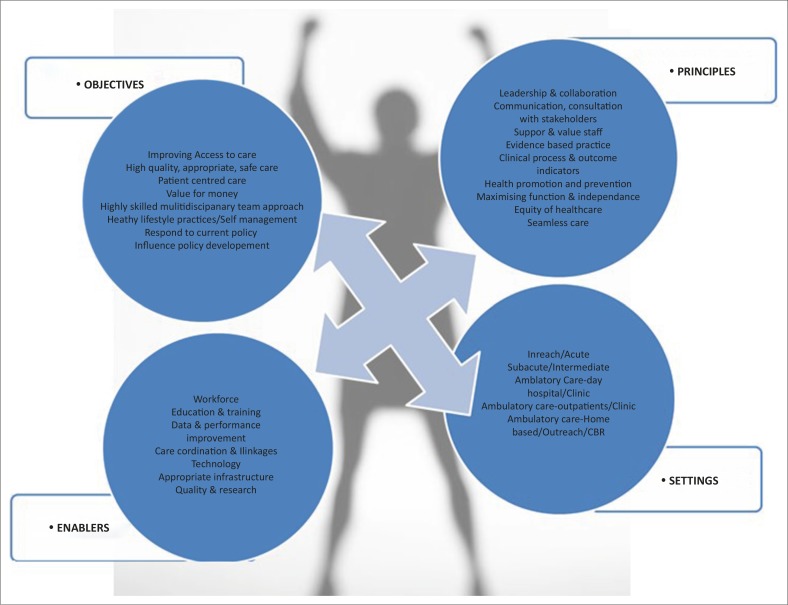
A synthesis of Australian models of care in rehabilitation.

The strategies involved in the development of the Australian rehabilitation models of care have been summarised and presented in a synthesis of Australian models of care in rehabilitation (Figure 1) (New South Wales Department of Health 2010; South Australia Department of Health 2011a; South Australia Department of Health 2011b; Western Australia Department of Health 2007; Western Australia Department of Health 2008). The models synthesis is explicit in addressing the rehabilitation needs identified to be lacking in a South African context. The trajectory of care for people living with HIV is linked with the care setting and underpinned by principles and critical enablers. The framework emphasises that the process of model development needs to include objectives (New South Wales Department of Health 2010). For instance the synthesised framework identifies the improvement of access to care, reducing inequality in health status, providing safe, high-quality health care; promoting a patient centred continuum of care; ensuring value for money and optimising health services as being part of the objectives driving the development of a model of care (New South Wales Department of Health 2010; South Australia Department of Health 2011a; South Australia Department of Health 2011b; Western Australia Department of Health 2007; Western Australia Department of Health 2008). At the same time this process needs to consider a number of principles such as leadership and collaboration of the multidisciplinary team, the specific setting is essential in providing appropriate timeous intervention. Furthermore, factors that will enable the implementation of a revised or new model such as data systems and education and training must be established during conceptualisation.

Reflections on working models enable researchers and health practitioners to identify gaps and causes of challenges within the system. For instance, the South Australia Department of Health (2011a) identified the need to develop a model of care for cardiac rehabilitation. It was evident that patients significantly benefited from rehabilitation programmes but many barriers existed that resulted in low participation such as local resource limitations (South Australia Department of Health 2011a), such resource limitations also impose themselves in our context, and these include fiscal challenges (Cobbing *et al.* 2014), which need to be factored into the development of a South African model. Likewise, a model of care for children with acquired brain injuries in Paris was implemented and although the evaluation rated the system to be organised, it lost a significant amount of children to follow up. It was discovered that the referral from the acute care hospital to long-term facilities such as outreach programmes and vocational guidance clinics was not always operational. In response informative documents were developed in order to strengthen adequate referral and follow-up (Chevignard *et al.* 2009). In South Africa, there is no model guiding rehabilitation of people living with HIV. The development of such a model could use elements of the Australian's guiding framework (see Figure 1) as a guiding tool. Drawing on the guiding framework will assist in identifying objectives, principles and the support needed in the South African context.

### Steps in the Development of a Model of Care

In order to guide the process of model development one has to identify logical steps and processes (Davidson *et al.* 2006). For instance, the Department of Health, Western Australia describes the Process of Developing a Model of Care in five major phases: Phase 1: understanding the health policy context, Phase 2: definition and understanding the current state of play, Phase 3: translating evidence-based research and expert opinion into best practice, Phase 4: consulting broadly with stakeholders and incorporating feedback, as appropriate to produce a finalised model of care, Phase 5: endorsement of the model of care by Advisory Group and Health Networks (Western Australia Department of Health 2007). These steps can also be used for the development of a rehabilitation model needed for people living with HIV in South Africa as it is explicit and provides comprehensive guidelines throughout the process of development. Propitiously, the current state of rehabilitation in public health care in South Africa fits into the framework and provides steps that can be adopted as the way forward in our paradigm.

Initial steps towards the development of such a model have commenced. For example, Phase 1: understanding the health policy context in South Africa is explored in preliminary work on HIV and disability. Evidence has been provided by Hanass-Hancock, Strode and Grant (2011) and Hanass-Hancock and Nixon (2010) revealing that current health policy does not include the disabling effects of HIV and its rehabilitation redress in HIV care in South Africa as yet. However, South Africa has developed a new National Strategic Plan (NSP) for sexually transmitted infections (STIs), HIV and TB for 2012–2016. The NSP‘s goals and strategic objectives are guided by evidence from various reports (South Africa 2012; South African National AIDS Council Disability Sector 2009; South African National AIDS Council 2011) and now includes the disability sector. The disability sector has responded to the challenge in developing disability specific HIV and AIDS programmes and established the need for mobilisation of resources for disability and prioritising persons with disabilities in the AIDS response (South African National AIDS Council Disability Sector 2009). The new NSP includes persons with disabilities as a vulnerable group and lists a number of services in relation to access, prevention, treatment care and support. This new plan is also dedicated to the management of HIV and AIDS and mentions the prevention of disability in the title of objective 3. Although, initial efforts are underway to integrate issues related to disability and HIV more needs to be carried out to concretely integrate a rehabilitation model to guide delivery of care. The plan does not include rehabilitation strategies such as physical, vocational and social approaches. Measurable outcomes need to be agreed upon and evaluated in order to assess the impact of these efforts on the broader goals of the NSP. In order to achieve integration rehabilitation has to be realised as a crucial component of HIV management in reducing disability (South African National AIDS Council 2011).

Secondly, Phase 2: definition and understanding the current state of play: Nixon (2011a) clearly describe the current state of rehabilitation in the context of HIV in South Africa highlighting the increasing disablement experienced by people living with HIV and association to the roll-out of ARTs in the mid-2000s. Cobbing *et al.* (2013), Hanass-Hancock *et al.* (2013) and Van As *et al.* (2009) concede and explain that as the number of people living with HIV increases in South Africa, the need to address their disabilities becomes an imperative on health care and health care professionals. However, strategies on streamlining intervention into the health structures remain a challenge.

Thirdly, Phase 3: translating evidence-based research and expert opinion into best practice: although South Africa is effectual in research pertaining to HIV and disability (Cobbing *et al.* 2013), much can be drawn from global contexts on the best practices and rehabilitation guidelines, such as the CWHGR (2013) e-module. This guide could be tailored to a South African context factoring in task shifting and a greater focus on community-based rehabilitation and home-based care. Consequently, some pilot projects (Cobbing *et al.* 2014; Petersen *et al.* 2014) indicate that rehabilitation (including mental health interventions) in the context of HIV in South Africa might need to go beyond standard of rehabilitation care which is often clinic based and limited because of a lack of qualified staff. Community-based rehabilitation and task shifting possibly provide a more feasible approach but this has not been discussed in the context of HIV and rehabilitation. Hence a broader consultative process working towards the development of feasible interventions is currently needed. Such a process needs to be discussed as possible models of care and feasible approaches for the South African context with experts and key stakeholders in the field. These experts and stakeholders should include the multidisciplinary health care team (doctors, physiotherapists, occupational therapists, dieticians, speech and language therapists, social workers, midlevel workers, community health care workers), department of health representative(s), site affiliated non-governmental organisation representative(s) and service users (people living with HIV receiving rehabilitation).

Consequently Phases 4 and 5: consulting broadly with stakeholders and incorporating feedback, as appropriate to produce a finalised model of care and endorsement of the model of care by Advisory Group and Health Networks are not yet initiated in South Africa. However, with expert opinions and reflection we will be able to develop evidence-based and feasible interventions. Only after this process, we will be able to agree on a model of care that is suitable to South Africa and that will be able to feed into the broader health agenda in South Africa. Such a model should involve communication both formal and informal in repetitive meetings to share information and solicit feedback regarding the sustainability and the running of the model. Furthermore, evaluation is often achieved by involving key stakeholders to give feedback on the progress and impact of the model (Cormack *et al.* 2007). This alludes to the pinnacle of this article, the way forward. The researchers highlight the necessity for a model of care in the rehabilitation in the context of HIV and that the process of developing this model needs to include consultative meetings with people living with HIV and service providers as well as consensus in feedback from experts in the field (Davidson *et al.* 2006).

## Conclusion

The need to develop a model to guide rehabilitation of people living with HIV in South Africa is essential as we address the cumulative disabling effects of the virus and its treatment. The process of development of the model needs to adhere to key processes that have already been tested in resource-rich contexts and now need to be further tailored to meet the needs of a resource poor context. A framework as in Figure 1 provides clarity on the elements that need to be considered in the development of such a model. Furthermore, the evidence shows that working models need phased development (Davidson *et al.* 2006; Western Australia Department of Health 2007). The example taken from the Western Australian Department of Health (2007) process of model development articulates seamlessly the phases that have consequently and ideally begun in South Africa through fundamental research (Cobbing *et al.* 2013). The upcoming processes will involve engagement with rehabilitation experts in the field of HIV and key stakeholders in order to obtain a guiding model of care in tackling the disabling effects of HIV on people living with the virus in South Africa.
